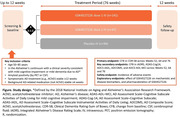# PROGRESS‐AD: a phase 2 study to evaluate efficacy and safety of GSK4527226 (AL101), an anti‐sortilin monoclonal antibody, in patients with early Alzheimer’s disease

**DOI:** 10.1002/alz.090247

**Published:** 2025-01-09

**Authors:** Sandra Machlitt‐Northen, Azza Abdelkarim, Christine Bailey, Balasubrahmanyam Budda, Stéphanie Bombois, Bruce Brew, Dimitra Brintziki, Brady Burgess, Sharon Cohen, Gopinath Ganji, Massimiliano Germani, Dave Inman, Tharaka Jayabalan, Catherine J. Mummery, Lovingly Park, Christine Parker, Gary Romano, Martin Sadowski, Giacomo Salvadore, Robert Y. K. Lai

**Affiliations:** ^1^ GSK, Stevenage, Hertfordshire United Kingdom; ^2^ GSK, Warsaw, Warszawa Poland; ^3^ GSK, Collegeville, PA USA; ^4^ Alector, South San Francisco, CA USA; ^5^ Hôpitaux Universitaires Pitié Salpêtrière, Charles Foix, Paris, Île‐de‐France France; ^6^ St Vincent’s Centre for Applied Medical Research, Sydney, NSW Australia; ^7^ GSK, Brentford, Middlesex United Kingdom; ^8^ Toronto Memory Program, Toronto, ON Canada; ^9^ GSK, Tres Cantos, Madrid Spain; ^10^ University College London, London, Greater London United Kingdom; ^11^ Drexel University, Philadelphia, PA USA; ^12^ New York University Langone Health, New York, NY USA

## Abstract

**Background:**

Progranulin (PGRN), a glycoprotein secreted by microglia and neurons, regulates lysosomal function, neuroinflammation, and has neurotrophic effects. Variants in the granulin gene (*GRN*) that cause a reduction of PGRN in plasma and cerebrospinal fluid (CSF) are associated with an increased risk of Alzheimer’s disease (AD). The sortilin receptor (SORT1) on neurons and microglia regulates PGRN degradation. GSK4527226 (AL101) is a human monoclonal immunoglobulin G1 antibody that inhibits SORT1 and increases extracellular PGRN levels in plasma and CSF. In Phase I studies, GSK4527226 was well tolerated with an acceptable safety profile in healthy volunteers, and showed dose‐dependent PGRN elevation in plasma and CSF (Ward et al. CTAD 2021; Germani et al. ACCP 2023).

**Method:**

PROGRESS‐AD is a phase 2, parallel group, randomized, double‐blind, global study which aims to evaluate the efficacy and safety of GSK4527226 versus placebo in patients with early AD (N = 282) (EU CT: 2023‐505083‐11‐00; NCT06079190). Eligible patients are in the Alzheimer’s continuum, with amyloid positivity and clinical severity consistent with mild cognitive impairment or mild dementia, as defined by the 2018 National Institute on Aging and Alzheimer’s Association Research Framework (**Figure**). Patients (aged 50‐85 years) will be randomized to receive intravenous GSK4527226 (dose 1), GSK4527226 (dose 2), or placebo for 76 weeks, followed by a 12‐week safety follow‐up period (**Figure**). Randomization will be stratified by APOE4 status, AD stage and participation in the CSF sub‐study. The primary endpoint is change from baseline (CFB) in the Clinical Dementia Rating‐Sum of Boxes (CDR‐SB) score across Weeks 52, 64, and 76. Key secondary endpoints include CFB in the iADRS, ADAS‐Cog14, ADCS‐iADL component of ADCS‐ADL‐MCI, and ADCOMS. Safety endpoints include incidence of treatment‐emergent adverse events. Key exploratory endpoints include effect of GSK4527226 on pharmacodynamic and disease biomarkers, and pharmacokinetics of GSK4527226. The primary analysis will use a proportional MMRM (Wang, 2022) under a Bayesian framework with a non‐informative prior distributions. Three sub‐studies will be conducted to assess the effects of GSK4527226 on amyloid PET, tau PET, and CSF biomarkers.

**Result:**

N/A.

**Conclusion:**

This study of the anti‐sortilin antibody, GSK4527226, will comprehensively evaluate its efficacy, safety and effects on pathogenesis in early AD.